# Cardiovascular magnetic resonance-derived aortic compliance, distensibility and pulse wave velocity at rest and during a supine bicycle exercise in young adults: A pilot study

**DOI:** 10.1186/1532-429X-16-S1-P171

**Published:** 2014-01-16

**Authors:** Laurence Bal-Theoleyre, Alain Lalande, Frank Kober, Monique Bernard, Alexis Jacquier

**Affiliations:** 1 Radiology, APHM, Marseille, France; 2 CEMEREM - CNRS 7339, Faculté de Médecine, Marseille, France; 3 Le2i - UMR CNRS 6306, Faculté de Médecine, Dijon, France

## Background

Purpose: Risk of aortic rupture is evaluated based on the vessel diameter; this parameter is probably insufficient. In vivo evaluation of biomechanical property of the aortic tissue might be of interest to discriminate between normal and altered aortic tissue (A Lalande et al, JMRI 2008). The purpose of this study was to describe a technique to measure regional aortic compliance (AC), aortic distensibility (AD) and aortic stiffness with 1.5 T MRI in young individuals under resting conditions and during supine bicycle exercise.

## Methods

Materials and Methods: Fiftheen young adults (8 men, median 29 (23-41) yrs) with no risk factor for atherosclerosis were recruited. They all underwent 1.5T MRI (Siemens, Avanto) at rest and during supine exercise using an amagnetic ergometer (Lode, the Netherlands). The following parameters were used for rest and exercise: cine = TR/TE = 30 ms/1.8 ms, thickness = 6 mm, α = 65°, matrix = 148 × 256, resolution = 30 ms, retrospective gating; Phase contrast = TR/TE = 25 ms/2 ms, thickness = 6 mm, matrix = 256 × 256, flow encoding = 200 cm/s, α = 25°, resolution = 25 ms, retrospective gating). For exercise the volunteer was instructed to cycle for 2 min at 25W, work load was increased each 2 min for a minimum of 10 min, to obtained twice the resting heart rate. The volunteer was instructed to stop cycling and immediately following which a breath held stress dataset of 2 acquisitions were acquired. The volunteer was instructed to cycle for at least 2 min at the last exercise level, and the images acquisition was repeated. AC and AD were determined from cine-MRI at four locations, ascending aorta (AA), proximal descending aorta (PDA), distal descending aorta (DDA), and aorta above renal artery (RA). Segmental aortic pulse wave velocity (PWV) was assessed by phase contrast.

## Results

**Figure 1:**
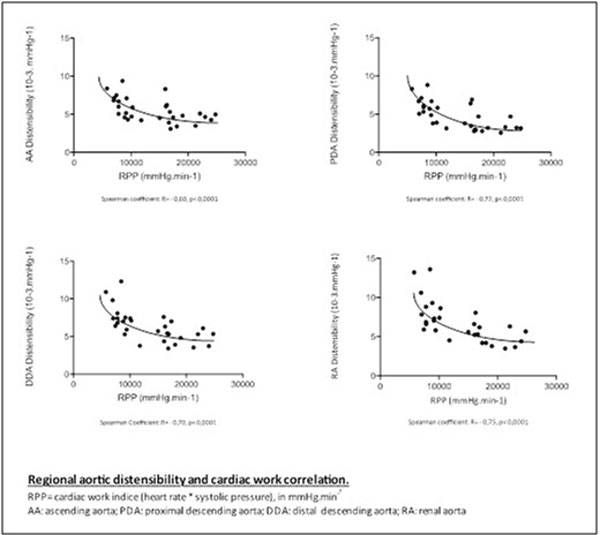


Results All volunteer complete the whole protocol. Stress induced a significant decrease of AC and AD at all sites (p <10-3). At rest and during stress, AC was statistically higher in AA compared to the whole descending aorta (p ≤0.0007). We found a strong correlation between the rate pressure product and AC at all sites (Figure 1). During exercise we measure a reduction of the aortic maximal diameter along the aortic arch (AA, p = 0.06 and PDA, p = 0.008) suggesting a contraction of the thoracic aorta during exercise. PWV measured at PDA and DDA increased significantly during stress (p = 0.02, p = 0.008, respectively).

## Conclusions

Conclusion This study demonstrates the feasibility to analyze regional aortic function during an exercise-induced stress MRI. Further studies are required to evaluate its interest to detect AA's dysfunction among patients with thoracic aortic diseases.

## Funding

Not applicable.

